# Emulating Perfection

**Published:** 1888-08

**Authors:** 


### EMULATING PERFECTION.

"*Palma non sine labore.*"

The above Latin quotation is here especially recommended to students of medicine, or those who have just entered the profession; and this article is written with the hope that it may encourage and animate to noble efforts this class of aspiring spirits in their chosen career.

It is notable that stagnation in any profession or business is fatal to progress and success. We never find Nature in a state of inertia, though the great majority of her forces are silent, and do their work of production and reproduction unheard and unseen. Yet in her vast laboratories and universal dominion, there is ceaseless activity going on from cycle to cycle, and from age to to age.

And the aim of Nature is perfection in each creation of its kind. No matter how humble or exalted the object, she expends upon it the best work of which she is capable, and fashions the lowliest flower, hiding its exquisite little face under the forest leaves, with as much painstaking and completeness as she does the loftiest mountains that lift their sublime fronts to the dome of the sky.

True men and women copy nature in emulating perfection, and take that soul inspiring word, "Excelsior" for their watchword through life. The pole star of a writer who has been indeed baptized with the divine afflatus, is perfection. The burning thoughts that spring up in his brain into instantaneous and glowing life are shaped again and again in his mind, carefully considered in all their relations, improved upon little by little, written out and re-written until they crystalize into words that thrill and exalt the mind of the reader, and perhaps give him an inspiration and impetus that will greatly aid in shaping his destiny through life.

The man who would be the architect of his own character aspires to have it perfect, and the true man in every condition of life desires to emulate perfection in his honorable labor, whether it be a work of the brain or hands.

What then are the requisites for emulating perfection in one's life work?

I would say first, a clearly defined perception of what that work is to be; next, let the man determine it he has a natural irrepressible inclination for the work, and therefore a fitness for its pursuit. A born poet is never a first-class mechanic; a farmer with a strong inclination for agricultural pursuits, cannot succeed as a doctor of law or medicine; and a man with a natural talent for merchandise, cannot have at the same time the immortal genius of an artist.

The natural bent of the man's mind being decided, then should follow noble aspirations to excel in the life-work he has chosen, and for which he is endowed by nature. Then there must be an indomitable resolution fixed in his mind and his purpose, that in his labor he will strive to reach the highest state of perfection human efforts can attain.

But while it is well to determine to do a thing, this will not avail unless the resolution is acted upon by the sledge hammer strokes of unflagging energy and perseverance, a giant in all undertakings, advances the labor with steady, persistent force, step by step, bravely disputing every inch of ground with all opposing obstacles.

One more important requisition is zeal, enthusiasm, a whole-hearted love for one's work, and this a man cannot have in its largest sense, unless he is fitted by nature for the calling. This full-souled love for one's work, makes it pleasant and grateful to the worker; it also inspires energy, force and other requisite agents for success and perfection.

When all of these are allied in a man's character and career and sublimely vitalized by a profound sense of that grand word, duty, which lays its kindly, but imperative hand, on every responsible man, and urges him to do some noble work through life—not for self alone, but for God and humanity—then he can be indeed, "a hero in the strife," and feel a reasonable assurance of sometime attaining in his labor that supreme height where he can truly and honestly say, "*Palma non sine labore.*" T. S. P.

